# Insights into WDR5: unveiling its functions, regulation, and impact on skeletal muscle

**DOI:** 10.1080/15592294.2025.2573998

**Published:** 2025-10-13

**Authors:** Erick Bahena-Culhuac, Mauricio Hernández-Somilleda, José Manuel Hernández-Hernández

**Affiliations:** Departamento de Genética y Biología Molecular, Centro de Investigación y de Estudios Avanzados del IPN, Ciudad de México, México

**Keywords:** WDR5, histone modifications, epigenetics, chromatin remodeling, muscle regeneration

## Abstract

WD40-repeat-containing protein 5 (WDR5) is a highly conserved multifunctional scaffold protein with a toroidal structure, facilitating interactions with numerous partners through its WDR5-binding motif (WBM) and WDR5-interacting (WIN) sites. It plays a critical role in histone modifications, including H3K4 methylation (H3K4me), histone acetylation, and deacetylation, influencing stem cell maintenance and differentiation. Recent studies highlight its involvement in muscle homeostasis, particularly in skeletal muscle progenitor cells, where it regulates PAX7-driven myogenic factor expression. Additionally, WDR5 governs epigenetic programs in smooth muscle by modulating H3K4me marks on lineage-specific genes. Despite extensive research on its role in cancer and chromatin remodeling, its broader physiological functions remain underexplored. This review examines WDR5’s regulatory mechanisms, including its modulation by long non-coding RNAs (lncRNAs), post-translational modifications (PTMs), and microproteins, while emphasizing its relevance to muscle biology. Understanding WDR5’s interactome and regulatory networks could provide novel insights into muscle regeneration, stem cell dynamics, and potential therapeutic strategies for muscular disorders and regenerative medicine.

## Introduction

WD40-repeat-containing protein 5 (WDR5) is also known as Cillia and Flagella Associated protein 89 (CFAP89), BMP-2 Induced Gene 3kb (BIG-3) or Set1c WD40 Repeat Protein (SWD3). WDR5 is a member of the WD40-repeat family found in eukaryotes, widely recognized for its functions as protein scaffolds, allowing the formation of multiple complexes, some of which are involved in gene regulation, signal transduction, and cell cycle progression [1].

WD40 repeats stands as a repetitive sequence motif of roughly 43–60 amino acids containing conserved residues, they were first identified in the CDC4 protein and in the Gβ subunit of heterotrimeric G proteins [2–4]. The name ‘WD40’ reflects the conserved WD motif and the length of each repeat [5–7]. As with many WD40 proteins, WDR5 originated from intragenic duplication and recombination events that gave rise to its β-propeller structure. Unlike other WD40 members that show considerable divergence among their repeats, WDR5 has retained a relatively high degree of conservation, both across its repeats and among different species. This evolutionary stability suggests that WDR5 emerged as a more specialized member of the family, with its conserved architecture reflecting strong selective pressure to maintain its role as a scaffold in chromatin-modifying complexes [1,8–10]

**Figure 1.**
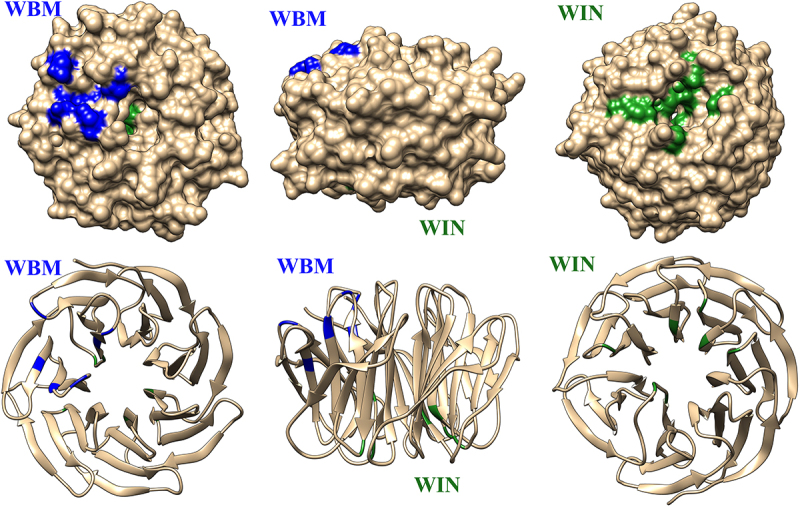
3D representation of WDR5. The WBM site is highlighted in blue, and the WIN site in green.

Notably, WDR5 exhibits over 90% sequence identity among vertebrates [11]. It has a distinctive feature that lies in its composition of 7 WD40 motifs, characterized by a series of 40 amino acid repeats, formed by Gly-His and Trp-Asp [12]. Importantly, each motif generates a β-propeller fold, with each blade consisting of a four-stranded antiparallel β-sheet [13]. This unique conformation imparts a toroid shape to WDR5, a critical aspect for its function. This shape is essential as it features a shallow cleft on one side known as the ‘WDR5-binding motif’ (WBM) site, and on the other surface, there is the ‘WDR5-interacting’ (WIN) site [10] (see Figure 1). These sites serve as the focal points where WDR5 engages in nearly 200 different protein interactions, either directly or indirectly [10], showing the relevance of this protein in different aspects of organogenesis and cell differentiation.

Given its versatile regulatory capacity, WDR5 also plays a role in maintaining muscle homeostasis. From a physiological standpoint, muscle homeostasis refers to the balance between catabolic and anabolic processes that regulate protein synthesis and degradation, enabling the muscle to maintain its mass and contractile functions [14]. These fundamental processes are tightly coordinated with epigenetic regulatory mechanisms, meaning that alterations in chromatin states or transcriptional control can directly affect muscle integrity and significantly impact the progression of various muscular disorders [15]. Conditions such as sarcopenia and some myopathies can compromise skeletal, cardiac, and smooth muscle integrity, reflecting shared molecular and physiological mechanisms [16–19]. Similarly, systemic syndromes such as cachexia, often associated with cancer or chronic inflammatory states, represent a widespread breakdown of muscle homeostasis that manifests as accelerated wasting, functional decline, and reduced quality of life, thereby highlighting the clinical relevance of deciphering the epigenetic mechanisms that safeguard muscle integrity [20–23].

Within this broader context, skeletal muscle is particularly interesting due to its high regenerative capacity, which depends on the proper function of muscle-specific stem cells, known as satellite cells (SCs) [24–26]. Post-natal SCs express high levels of the Paired-Box 7 (PAX7) transcription factor, whose activity promotes transcriptional programs that allow the progression of adult myogenesis. In this context, PAX7 recruits WDR5 and the MLL complex at gene regulatory regions of Myogenic Factor 5 (Myf5). These events correlate with chromatin accessibility, enrichment of H3K4me3 at this locus and induction of Myf5 mRNA [27,28]. Hence, H3K4 methylation emerges as an important feature associated with WDR5 function, since smooth muscle lacking expression of WDR5 substantially reduces H3K4me levels at specific smooth muscle cell genes promoters. These observations suggest a mechanism of how this protein play a crucial role on activation of muscle cell lineage genes [29]. Nevertheless, due to the vast diversity of WDR5 functions, additional regulatory mechanisms remain largely unexplored. While much of the existing research has focused on developing small-molecules to inhibit WDR5 in cancer, this review aims to provide a comprehensive overview of its multifaceted roles across a different cellular environment, exploring key proteins that interact with WDR5, their cooperative functions and regulatory mechanisms in muscle biology, with an emphasis on skeletal muscle.

## Epigenetic regulation by WDR5

WDR5 is a versatile epigenetic regulator that plays a central role in chromatin modification. It forms complexes with MLL/SET1 to catalyze H3K4 methylation, a mark typically associated with gene activation [30]. However, WDR5 is not exclusively linked to this; it can also associate with repressive complexes such as NuRD, contributing to transcriptional silencing [31]. This dual functionality allows WDR5 to participate in diverse chromatin-modifying assemblies, mediating methylation, acetylation, and deacetylation depending on cellular context. Such versatility underscores its critical role in orchestrating gene expression programs across different tissues and conditions, making WDR5 a key focus in studies of epigenetic regulation [32,33].

### H3K4 methylation

WDR5 plays a pivotal role in several cellular functions, primarily centered around histone modifications (Table 1). One of its prominent functions lies in its integral involvement as a core component of the WRAD complex, comprised of Retinoblastoma Binding Protein 5 (RbBP5), Absent-Small-Homeotic-2-Like protein (ASH2L), and Dumpy-30 protein (DPY30) [65]. Through its association with the SET1/MLL family of histone methyltransferases (HMTs), WRAD promotes methylation of the fourth lysine residue on histone H3 (H3K4), a modification crucial for transcriptional programs and the maintenance of accessible chromatin state [10].

**Table 1.** WDR5-interacting proteins and their collaborative functions.

Protein Associated	Union Site	Function		Reference
RbBP5	WBM	Formation of the WRAD complex		[34,35]
MLL1	WIN	H3K4 methylation		[36–38]
MLL2	WIN			[36,39]
MLL4	WIN			[36,40]
SET1A	WIN			[36,41]
SET1B	WIN			[36]
H3	WIN	Histone Tail Reader	H3R2me2s	[42,43]
			H3R2me1	[43]
			H3Q5ser	[44]
			H3K4me2	[37,45,46]
			H3T11P	[47]
lncCARs	WBM	Promote gene expression within Divergent Transcription promoters		[48]
MBD3C	WIN	Formation of the NuRD complex		[49,50]
CHD4	–			
MTA2				
RBBP4				
HDAC2				
	–	H3K4 deacetylation, of PHD2 promoter		[51]
Atrophin 2	–	Formation of WHHERE complex		[52]
ING2	–	Inhibition of γ-globin expression		[53]
HDAC1				
PRMT5				
HDAC3	–	Regulation of the hypoxia-induced EMT		[54]
KANSL1	WIN	Forming NSL complex		[55,56]
KANSL2	WBM			
ESC lncRNAs	WBM	Stem cell pluripotency		[57]
HOTTIP	–	Activation of the HOXA gene cluster		[58]
INO80	–	Positive regulation of the Wnt signaling transduction		[59]
MERRICAL	–	Activation of CCL3 and CCL4		[60]
DBE-T	–	DUX4 induction in FSHD		[61]
ANRIL	–	Formation of HDAC3-WDR5 complex		[62]
Pask	–	MuSC activation and differentiation		[32]
APC/C	WIN	Swift expression of pluripotency genes		[63]
TBP	WBM			
EMBOW	WIN	Regulates WDR5´s WIN site interaction		[64]

The WRAD complex assembles with a defined stoichiometry of 1 WDR5, 1 RbBP5, 1 ASH2L, and 2 DPY30. Recent cryo-EM studies by Rahman et al., 2022 indicate that WRAD recruitment occurs through a stepwise pathway orchestrated by individual subunits [66]. Initial binding is mediated by RbBP5, whose WD40 domain engages the nucleosome, providing the primary anchoring interface. WDR5 is then recruited, forming a stable density alongside RbBP5 and stabilizing the catalytic SET domain of MLL1. Partial integration of MLL1 and ASH2L follows, with ASH2L extending its intrinsically disordered and SPRY regions toward nucleosomal DNA. Finally, the DPY30 homodimer binds ASH2L via its SDI motif, stabilizing the complex and promoting higher-order H3K4 di- and tri-methylation. This sequential assembly ensures that each subunit contributes both structurally and functionally, with WDR5 serving as a central scaffold that directly interacts with RbBP5 through its WBM site and with MLL1 via the WIN motif, enabling the formation of a fully assembled, catalytically competent complex [34–36,67]. Notably, two aromatic residues, Phe133 and Phe263, are critical for stabilizing WDR5’s interaction with SET1/MLL enzymes [68,69], emphasizing how precise subunit contacts are essential for WRAD-mediated H3K4 methylation.

In skeletal muscle, H3K4 methylation underlies the activation of key myogenic genes during development and regeneration, supporting chromatin accessibility and lineage-specific transcription [27,28]. H3K4 exist in three methylation states, mono-(H3K4me1), di-(H3K4me2), and tri-methylated (H3K4me3), all of which are associated with euchromatin and gene expression [70]. H3K4me3 is typically enriched at transcription start sites (TSS) where it facilitates the recruitment of RNA-polymerase II (Pol II), promoting transcription initiation [45,70], whereas H3K4me2 spans broader gene body regions and H3K4me1 localizes primarily at distal enhancers [45,70]. Notably, WRAD alone is capable of promote H3K4me1, but this activity is confined to the H3/H4 tetramer [65,71]. Thus, full methylation requires the concerted action of both WRAD and SET1/MLL proteins.

**Figure 2.**
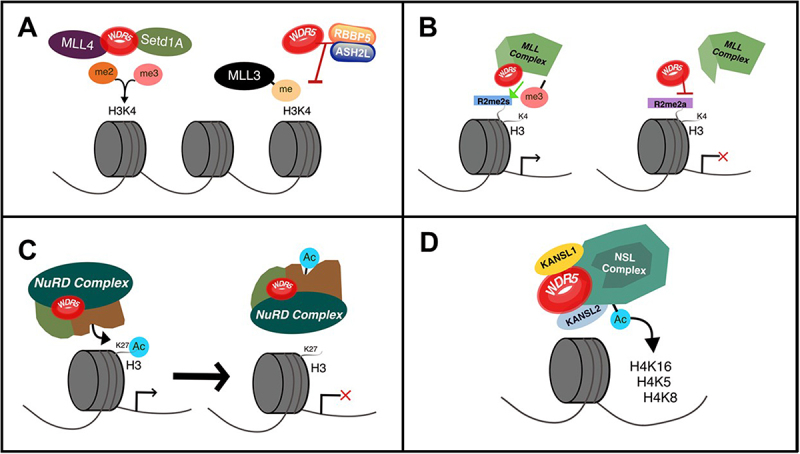
Epigenetic regulation by WDR5. A) Complex with MLL4 and SETD1A lead to H3K4 di and trimethylation. Interaction with RbBP5 and ASH2L block MLL3 activity. B) Recognition of histone modification like R2me2s and R2me2a. C) Histone deacetylation of H3K27 with the NuRD complex. D) Histone acetylation of H4K16, H4K5 and H4K8 with the NSL complex.

The SET1/MLL family comprises six distinct members: MLL1-4, SET1A, and SET1B [72]. WDR5 modulates the activity of each complex member, generally enhancing their enzymatic functions to varying extents. An intriguing exception is MLL3, where the loss of WDR5 actually increases its monomethyltransferase activity [36,73,74]. This effect appears to be mediated either through disruption of the RbBP5/Ash2L interaction [73] or independently of it [36]. Interestingly, despite this functional divergence, the WIN motif of MLL3 retains high-affinity binding to WDR5 [73] suggesting that WDR5 May act as a negative regulator of MLL3 activity (Figure 2(A)). In contrast, WDR5 positively influences the activities of MLL1, MLL2, MLL4, SETd1A, and SETd1B, although it exerts distinct effects on each complex. Specifically, MLL1 and SETd1A complexes rely entirely on WDR5 for their complete di- and trimethylation activities [74], highlighting the critical role of WDR5 in their assembly and function (Figure 2(A)). Disruption of the WIN motif within WDR5 significantly impairs the assembly and activity of these complexes [75]. Conversely, WDR5’s role in MLL2/4 and SETd1B displays greater variability. While some studies suggest a crucial role for WDR5 in these complexes [36,39,40,76] others indicate only modest involvement [74,75,77]. This discrepancy may be more closely related to the selective modulation of WDR5 toward specific methylation states, as many studies do not differentiate between mono, di, and trimethylation. However, research focused on distinct methylation marks has provided clearer insights. For example, in Kaposi Sarcoma, the H3K4me3 mark has been shown to depend on MLL2 and WDR5 in complex with glutamate-rich WD repeat containing 1 (GRWD1) [39]. Similarly, in pancreatic cancer, MLL4 in complex with WDR5 is essential for establishing the H3K4me1 mark and promoting tumor progression [40].

### Recognition of histone modifications

WDR5 exhibits a notable capacity to recognize specific histone modifications (Table 1), influencing chromatin accessibility and gene expression regulation [10,78]. Among these, its interaction with methylated arginine 2 on histone H3 (H3R2) is particularly well characterized. Asymmetric di-methylation (H3R2me2a) inhibits WDR5 binding, thus preventing H3K4 methylation by the SET/MLL complex, resulting in transcriptional repression [42,78]. In contrast symmetrical di-methylation (H3R2me2s) supports WDR5 recruitment and co-localizes with active chromatin marks H3K4me1 and H3K4me3 near promoters [42,43]. The methylation interchange process is meticulously governed by protein-arginine methyltransferases (PRMTs), PRMT6 deposits H3R2me2a, whereas PRMT5 and PRMT7 generate H3R2me2s and H3R2me1, respectively [42,43,79] (Figure 2(B)). Notably, PRMT5 and PRMT7 also play essential roles in the regulation and regenerative capacity of skeletal muscle satellite cells (SCs) [80–82], suggesting a potential indirect role for WDR5 in myogenesis through arginine methylation pathways.

WDR5 also recognizes serotonylation of glutamine 5 on histone H3 (H3Q5ser) and H3K4Me2 (Table 1) [37,83]. H3Q5ser is catalyzed by Transglutaminase 2 (TGM2), that is often found in combination with H3K4me3. This dual modification plays a significant role in the transcriptional activation of genes by enhancing the recruitment of transcription factor IID (TFIID) [83,84] Particularly, H3Q5ser facilitates the recruitment of WDR5 to the chromatin contributing to transcriptional activity [83]. Mutations in WDR5’s WIN site reduces the H3K4me3 level in neuroblastoma cells, possibly through disrupted recognition of modified H3 tails [83]. However, H3Q5ser has been shown to have no effect on MLL1 activity [44]. In this way, H3K4me3 decrease might be related to WDR5 working alongside another member of the SET1/MLL family.

On the other hand, while WDR5 has demonstrated its ability to bind with the H3 tail, several studies indicate a higher affinity towards H3K4me2 [37,45,46]. Notably, the only reported interaction between WDR5 and H3K4me2, unrelated to WDR5’s influence on establishing the H3K4me2 mark, is their interaction with active chromatin associated lncRNAs (lncCARs) within Divergent Transcription (XH) promoters, inducing gene expression [48]. However, it is important to note that the binding between WDR5 and H3K4me2 has not been examined. Nevertheless, lncCARs were observed to interact with the WBM site (Phe266) of WDR5, suggesting a potential direct interaction between WDR5’s WIN site and H3K4me2 [48]. Furthermore, Subhash et al. hypothesize that in the context of XH, lncCARs/WDR5 is essential for the conversion of H3K4me2 to H3K4me3. In skeletal muscle, regions primed with H3K4me2 are key for activating myogenic genes like Myf5 and MyoD during satellite cell activation, a process regulated by Pax7 and SET1/MLL complexes [85,86], raising the possibility that WDR5 could support this epigenetic transition [87]. Further investigation into WDR5’s binding preferences and dynamics at H3K4me2-enriched loci in muscle stem cells could reveal a broader role for WDR5 in regulating transcriptional priming during myogenic commitment.

Beyond methylation-associated interactions, WDR5 also demonstrates versatility in histone mark recognition. For instance, it has been shown to recognize threonine 11 phosphorylation on histone H3 (H3T11P), a mark involved in androgen receptor signaling. This highlights WDR5’s broader role in interpreting combinatorial histone codes to recruit SET1/MLL complexes and drive gene expression in a context-dependent manner [47].

Interestingly, recent findings have extended the relevance of H3T11 phosphorylation (H3T11P) to skeletal muscle differentiation. A study by [88] demonstrated that during myogenic commitment, the glycolytic enzyme PKM2 translocates to the nucleus and phosphorylates H3T11 at promoters of myogenic regulatory genes such as Myog and Myh3. This phosphorylation event was shown to be crucial for chromatin accessibility and transcriptional activation during myoblast differentiation. Given that WDR5 recognizes H3T11P [47], it is plausible that WDR5 could interpret this modification to recruit SET1/MLL complexes and promote H3K4 methylation at muscle-specific gene loci, suggesting a coordinated mechanism by which WDR5 couples metabolic signals to epigenetic remodeling during muscle development.

### Histone deacetylation

In addition to methylation, WDR5 has also been extensively reported to be part of deacetylase complexes [10] (Table 1), notably the ATP-dependent Nucleosome Remodeling and Deacetylase (NuRD) complex [89], serve as a transcriptional co-repressor, intricately modulating both gene silencing and activation processes [89]. The diversity of NuRD complexes is underscored by its various combinations, each exhibiting distinct activities. Notably, within the realm of pluripotency, the NuRD-Polycomb Repressive Complex 2 (PRC2) and NuRD-Cdk2ap1 complexes have garnered significant attention [49]. NuRD’s pivotal role in gene regulation is further emphasized by its ability to deacetylate H3K27, facilitating its subsequent trimethylation by PRC2 at bivalent promoters [49,90]. Moreover, the interplay between Cdk2ap1 and NuRD is crucial for cellular differentiation [49,91]. Also, both NuRD complex variants are known to interact with WDR5 [49]. Recent studies have shed light on WDR5’s affinity for a member of the NuRD-Cdk2ap1 complex, the less abundant isoform of MBD3, MBD3C, interacting with WDR5 at the WIN binding site [31,50].

Moreover, WDR5 shows the capacity to interact with other key components of the NuRD complex, including Chromodomain-helicase-DNA-binding protein 4 (CHD4), Histone deacetylase 2 (HDAC2), Metastasis-associated protein 2 (MTA2), and Retinoblastoma-binding protein 4 (RBBP4) [49]. Therefore, WDR5/NuRD complexes play a vital role in pluripotency and differentiation (Figure 2(C)).

Furthermore, the NuRD complex is a key regulator of chromatin architecture and gene expression during skeletal muscle regeneration. It has been shown to maintain satellite cell identity by repressing non-myogenic lineage genes, thereby ensuring proper myogenic progression [92]. Disruption of NuRD components, such as CHD4, leads to impaired regeneration and lineage infidelity, underscoring its critical role in safeguarding muscle stem cell fate. In pathological contexts like critical illness myopathy, disassembly of the NuRD complex in human SCs correlates with impaired repair capacity [93]. Given the crucial role of the NuRD complex in muscle, it is possible that WDR5 May contribute to muscle-specific NuRD functions by modulating chromatin accessibility or recruiting additional epigenetic factors.

WDR5 also interacts with histone deacetylases (HDACs) in various biological contexts (Table 1), underscoring its versatility to form different complexes. One example is the formation of the WHHERE complex with HDAC1, HDAC2, and the transcriptional co-regulator RERE (Atrophin-2), which is critical for retinoic acid signaling and proper somite symmetry during development [52]. Notably, within this complex, WDR5 interacts directly with Atrophin-2 but not with the HDACs themselves [52].

In contrast, direct binding between WDR5 and HDAC1 has been reported in the regulation of γ-globin gene expression. In this setting, WDR5 interacts with PRMT5 to promote H3K4 trimethylation at the γ-globin promoter, a modification that subsequently recruits ING2 and HDAC1. This cascade leads to histone deacetylation and transcriptional repression, illustrating a functional shift from gene priming to silencing through coordinated histone modifications [53,94]. Additionally, WDR5 forms a complex with HDAC3 in the context of hypoxia-induced epithelial-mesenchymal transition (EMT). Here, HDAC3 directly associates with WDR5 to facilitate the recruitment of methyltransferase complexes to H3K4, contributing to HIF-1α-driven transcriptional programs during EMT and metastasis [54].

### Histone acetylation

WDR5 plays a significant role in histone acetylation complexes as well (Table 1). The most well-reported interaction is within the non‐specific lethal (NSL) complex [10], composed by the histone acetyltransferase Males absent on the First (MOF) and another 2 subunits KAT8 Regulatory NSL Complex Subunit 1 (KANSL1) and KAT8 Regulatory NSL Complex Subunit 2 (KANSL2). WDR5 forms direct associations with both of them to acetylate H4K16, H4K5, and H4K8 [95]. Notably, KANSL1 binds to the WIN site of WDR5, while KANSL2 interacts with the WBM site [55] (Figure 2(D)). Despite the apparent contrariety between NSL and histone methyltransferase (HMT) complexes, it is intriguing to note that the NSL complex has been reported to induce the activity of SET1/MLL complexes by an acetylation mechanism, leading to H3K4 di-methylation and subsequent gene activation [56]. However, whether WDR5 facilitates this recruitment remains a mystery, warranting further investigation.

WDR5 interacts with the Double-Histone-Acetyltransferase (ATAC) complex and lysine acetyltransferase 2B (KAT2B), both critical for chromatin remodeling and transcriptional regulation. The ATAC complex, essential for development and cell cycle control, includes acetyltransferases GCN5 and ATAC2, which are associated with WDR5 alongside other components such as MBIP and DR1 [96]. KAT2B, a histone acetyltransferase, also interacts with WDR5 to regulate metabolic gene expression through glucagon-induced H3K9 acetylation and transcriptional activation via the CREB – CRTC2 axis [97]. This has particular relevance in skeletal muscle, where KAT2B has been shown to promote myoblast proliferation by enhancing the expression of Ccna2, Cdc25c genes, involved in the cell cycle [98]. Although a direct link between WDR5 and KAT2B in muscle has not yet been demonstrated, the known interactions of WDR5 with both acetyltransferases and methyltransferases, together with the essential role of KAT2B in muscle differentiation, suggest a potential epigenetic axis through which WDR5 could modulate chromatin structure during myogenesis. Thus, WDR5 May act as an integrative hub, coordinating acetylation and methylation dynamics to fine-tune transcriptional programs in skeletal muscle (Bode et al., 2016; Guelman et al., 2009; Suzuki et al., 2018).

As WDR5 interact with chromodomain-containing partners such as CHD4, little is known about its potential associations with bromodomain-containing proteins, which act as key readers of acetylated lysines and mediate transcriptional activation [49].

To date, the reported context is in melanoma cells, where co-immunoprecipitation studies suggest that WDR5 can form a complex with BRD4 via the lncRNA NEAT1, maintaining both proteins in a low-activity state [99]. Outside of this context, direct interactions between WDR5 and bromodomain proteins have not been validated, and their functional relevance remains an open question. Investigating this axis in other cell types could reveal whether WDR5 cooperates with bromodomain readers to integrate methylation and acetylation signals in chromatin regulation.

## Regulation of WDR5

### LncRNAs

**Figure 3.**
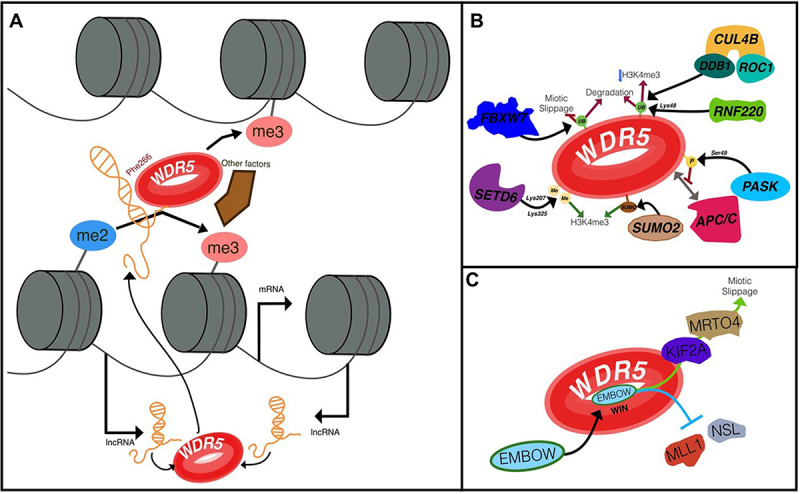
Known mechanisms of WDR5 regulation. A) LncRnas can guide WDR5 by interacting around Phe266, facilitating its recruitment across the genome and promoting H3K4me3 deposition with the help of other factors. This regulation occurs in a cis or trans manner. In the case of antisense lncRnas, the process begins with the recognition of H3K4me2. B) Known PTMs of WDR5. Ubiquitination by FBXW7, RNF220, and CUL4B/DDB1/ROC1 triggers the degradation of WDR5. FBXW7 inhibits mitotic slippage, while the others reduce H3K4me3. Methylation of Lys207 and Lys325 by SETD6, or SUMOylation by SUMO2, stimulates H3K4me3 deposition, while phosphorylation of Ser49 by PASK alters its interactome, for example by inhibiting its interaction with APC/C. C) the microprotein EMBOW regulates WDR5 by interacting within the WIN site, altering its interactome by promoting interactions with KIF2A and MRTO4, thereby enhancing its role in mitotic slippage, while inhibiting interactions with chromatin regulators such as MLL1 and NSL.

According to previous evidence, WDR5 plays a diverse range of roles and engages in various interactions. Certain complexes associated with WDR5 May promote gene expression, while others may suppress it. Furthermore, research has highlighted WDR5’s significance in both cell differentiation and pluripotency, indicating the likelihood of its regulation or guidance through specific mechanisms. With this in consideration, in the last couple of years lncRNAs have emerged as promising candidates for guiding WDR5 through the genome. As discussed earlier, Subhash et al., classified 209 lncRNAs as active chromatin associated RNAs due to their evident role on gene transcription (Table 1) [48]. Moreover, 43% of those lncRNAs were related to divergent transcription units. In this context, antisense lncRNAs such as FOXD3-AS1, HOXC13-AS, and GATA6-AS1, play a crucial role in positively regulating the expression of their respective counterparts: FOXD3, HOXC13, and GATA6 genes. This regulation occurs through the recruitment of WDR5, which facilitates the conversion of H3K4me2 to H3K4me3, thereby establishing transcriptionally competent chromatin at these loci (Figure 3(A)) [48].

Another antisense lncRNA, Brain-derived neurotrophic factor (BDNF-AS) has been reported to regulate ferroptosis on gastric cancer (GC) through WDR5 recruitment at WD repeat domain-containing 7 (FBXW7) promoter, facilitating the H3K27me3 repressor mark and decreasing its expression. This leads to a deficient ubiquitination of voltage-dependent anion channel 3 (VDAC3) and subsequent ferroptosis [100]. It is noteworthy that in the same year Hänle-Kreidler, et al. reported that WDR5 can be a substrate of FBXW7 leading to its ubiquitination and subsequent degradation. This is very interesting as in the context of GC ferroptosis it could be part of a regulatory loop mechanism where less levels of FBXW7 avoid WDR5 degradation and leads to a continuous ferroptosis, but further investigation is needed [101].

Furthermore, lncRNAs play a critical role in maintaining WDR5’s function in stem cell pluripotency [57], Yang et al. identified Phe266 as crucial for the interaction between lncRNAs and WDR5, a finding supported by Subhash et al (2018). This residue serves as the primary site of WDR5 interaction with lncRNAs. Remarkably, the mutation of Phe266 resulted in an 80% reduction in H3K4me3 levels [57], while leaving WDR5’s interaction with MLL1, H3, RbBP5 or Ash2L unaffected, despite these proteins bind to the WBM region where Phe266 is located (Table 1) [57]. However, this mutation selectively impacted WDR5’s interaction with 1434 RNAs, including mRNAs, lncRNAs, pri-miRNAs, and snoRNAs [57]. Consequently, there was a significant reduction in mRNA levels of pluripotency regulators such as Nanog or Sox2, which expression have been previously reported to be promoted by WDR5 [102,103]. Among these lncRNAs, WDR5 was found to specifically bind to 23 previously identified ESCs lncRNAs, including lincRNA-1552 and lincRNA-1592 [57].

Furthermore, more specific interactions between a single lncRNA and WDR5 have been also reported. One notable example is the relationship between WDR5 and the HOXA transcript at the distal tip (HOTTIP). Positioned at the 5′ tip of the HOXA locus, HOTTIP is an antisense lncRNA [104]. Widely implicated in various cancers, this lncRNA plays a significant role [104]. HOTTIP directly binds to WDR5, facilitating H3K4 induction through WDR5/MLL complexes across the HOXA gene cluster. This interaction triggers H3K4me3 mark, promoting gene transcription [58]. Remarkably, depletion of HOTTIP RNA results in the downregulation of distal HOXA genes, including HOXA13, HOXA11, HOXA10, HOXA9, and HOXA7 [58]. Furthermore, HOTTIP’s role in regulating HOXA9 expression has been independently reported [105]. Within the context of prostate cancer, TWIST1 upregulates Hoxa9 through its interaction with WDR5 and HOTTIP [105].

HOTTIP has been widely described in the context of cancer, but it is also crucial for neuronal development. Notably, WDR5 or HOTTIP deficiency disrupts the apical and basal polarity of dendrites and reduces the expression of Reelin signal receptors like ApoER and VldlR, whose expression is linked to epigenetic deregulation and abnormal histone marks at their promoters (Ka et al., 2022). It was described that HOTTIP form complexes in association with ASH2L and KANSL1 proteins mediated by WDR5 in cortical neuron cells. Finally silencing of HOTTIP *in utero* into E14.5 embryos resulted on abnormal dendrite polarity and orientation in cortical pyramidal neurons, demonstrating not only the importance of WDR5 but also its lncRNA binding partner HOTTIP during development [33].

Furthermore, other lncRNAs have emerged as significant regulators of WDR5 activity within other physiological context. For instance, the lncRNA Macrophage-enriched lncRNA (MERRICAL) orchestrates the regulation of chemokines CCL3 and CCL4 by guiding WDR5 to the respective loci, thereby promoting H3K4me3 [60]. Similar to a -*cis* regulation of D4Z4 binding element-transcript (DBE-T) of double homeobox 4 within Facioscapulohumeral muscular dystrophy (FSHD) [61]. In contrast, the lncRNA ANRIL acts as a scaffold, facilitating the formation of WDR5 and HDAC3 complexes [62,106]. While most lncRNAs are known to guide WDR5, the unique ability of ANRIL to promote the formation of a specific complex is particularly noteworthy. The implications of these activities within the smooth muscle context will be further explored in section 5. However, the remarkable role of lncRNAs in modulating WDR5 activity promises a novel approach to gene regulation. Especially intriguing is the increasing evidence suggesting that lncRNAs can travel within extracellular vesicles (EVs) [106]. Hence, the modulation of WDR5 activity via lncRNAs transported by EVs stands as a compelling avenue for further investigation and exploration.

### Posttranslational modifications

Post-translational modifications (PTMs) are pivotal in governing protein activity, stability, and interactions. Whether it occurs on single or multiple residues, they intricately coordinate to shape overall protein function [107,108]. In this way, due to the multifaceted role of WDR5, PTMs could be a key factor within WDR5 activity, especially within the association within multiple complexes (Figure 3(B)). However, it’s noteworthy that to this point, there are not many reported PTMs associated with WDR5. WDR5´s ubiquitination is the best reported PTM. One of the pivotal regulators in orchestrating WDR5 ubiquitination and its subsequent degradation is F-box and WD repeat domain-containing 7 (FBXW7) (Figure 3(B)). This is particularly crucial during mitotic arrest. Usually, cells utilize a mechanism known as mitotic slippage to evade cell death, with WDR5 playing a significant role in this process. Hence, FBXW7, recognized as a tumor suppressor, prevents mitotic slippage by mediating the ubiquitination of WDR5 [101]. Another critical regulator of WDR5 ubiquitination is Cullin-4B (CUL4B), which is recruited by Damage Specific DNA Binding Protein 1 (DDB1) (Figure 3(B)). This complex directs WDR5 to the CUL4B-ROC1 E3 ligase, specifically within the nucleus, for ubiquitylation (Nakagawa & Xiong, 2011). Notably, this process has been observed in the context of nerve growth factor (NGF)-induced neurite extension in PC12 cells [109]. The negative modulation of H3K4me3 levels by CUL4B through WDR5 degradation plays a crucial role in regulating the expression of neuronal genes [109]. Neither complex highlights the ubiquitination of WDR5 within a specific region or residue. Recently it was noted that another E3 ubiquitin ligase, Ring Finger Protein 220 (RNF220) is also capable of inducing WDR5 polyubiquitylation and degradation (Figure 3(B)). Causing the downregulation of Hox genes in the context of neuronal development. In this case, Lys48 was defined as the residues that harbor the polyubiquitination mark for proteasomal degradation [110].

In addition to its ubiquitination, WDR5 has also been found to undergo methylation on specific lysine residues (Figure 3(B)). Specifically, lysines 207 and 325 of WDR5 have been identified as targets for monomethylation by SET domain-containing protein methyltransferase 6 (SETD6). On one hand, Lys207 locates within WBM region, whereas Lys325 locates within the WIN site. In breast cancer cells, this methylation event has been associated with the promotion of cell proliferation and migration [111]. While the methylation of Lys207/Lys325 on WDR5 contributes partially to the maintenance of global histone trimethylation at lysine 4 on histone H3, it does not seem to impact the assembly of the MLL/SET1 complex [111]. However, further investigations are necessary to determine whether this methylation affects various aspects of WDR5 functionality, including its histone reading ability, acetylation, deacetylation, interaction with transcription factors, or other roles.

Another PTM also reported to modify WDR5 is SUMOylation (Figure 3(B)) [112]. has reported that over SUMOylation by in vitro assays and also through ectopic expressions in HeLa cells, RbBP5 and WDR5 can be a target of SUMO2, detected by SUMO2/3 antibody. Hence, it was proposed that this modification could be a mechanism to modulate the subunit assembly of SET1/MLL complexes and consequently the H3K4 methylation through demodification catalyzed by SUMO Specific Peptidase 3 (SENP3) in the context of osteogenic differentiation [112]. This is the only report so far about WDR5 SUMOylation. It is noteworthy that this modification was only observed in these specific conditions, and this has not been already described in another context.

Finally, the most well described PTMs is the Ser49 phosphorylation via PAS domain-containing Kinase (PASK) (Figure 3(B)) [32]. In the realm of stem cell and muscle stem cell activation and differentiation, the phosphorylation of Ser49 or the introduction of an S49E mutation has been found to dampen the interaction between WDR5 and the anaphase-promoting complex/cyclosome (APC/C), leading to a loss in the cells’ ability to sustain self-renewal [113]. While WDR5 engages with APC/C through the WIN site, its binding with the TATA binding protein (TBP) occurs at the WBM site [63]. This complex is directed to specific transcription start sites (TSS) during mitosis, facilitating APC/C in marking histones for degradation. This ensures swift expression of pluripotency genes in the subsequent cell cycle [63]. The repercussion of this change within the muscle will be further discussed on section 4.1.

### EMBOW

An interesting molecule, known to modulate WDR5 activity is a micropeptide called endogenous microprotein binder of WDR5 (EMBOW) (Figure 3(C)) [64]. EMBOW, originating from a uORF within the SCRIB gene, directly interacts with WDR5 at its WIN site, thereby shaping WDR5’s WIN interactome. Elevated EMBOW levels enhance the binding of KIF2A and MRTO4 to WDR5 while reducing associations with MLL1 and other factors, such as those in the NSL complex [64]. Notably, both KIF2A and MRTO4 are critical for the mitotic spindle, suggesting that EMBOW drives a transition in WDR5’s function from an epigenetic regulator to a structural component within the mitotic spindle. Furthermore, EMBOW expression peaks during late G1 phase and G2/M phase, whereas its depletion heightens the interaction between MLL1 and WDR5, resulting in off-target H3K4me3 generation [64]. Thus, EMBOW finely balances WDR5’s engagement in epigenetic modifications and its contribution to the mitotic spindle, ensuring proper cellular function across various phases of the cell cycle.

## WDR5 on skeletal muscle

Due to the intrinsic role of WDR5 within pluripotency and differentiation, it is not unexpected that WDR5 also plays a crucial role within skeletal muscle homeostasis and regeneration. The most well-reported role is within Skeletal Muscle Satellite Cells (SCs) differentiation [114]. SCs are adults stem cells that, once activated upon stimuli, start a myogenic program of differentiation to rebuild skeletal muscle [115]. Each cell state is controlled by specific factors, as SCs are controlled primarily by Paired Box 7 (Pax7). Differentiating myoblasts are controlled by multiple myogenic factors such as: Myogenic factor 5 (Myf5), Myogenic Differentiation 1 (MyoD), and Myogenin (MyoG) [115,116]. WDR5 has shown to have a direct effect on Pax7, Myf5, and MyoG [27,32,113].

### PASK-WDR5

WDR5 dynamically regulates the expression of Pax7 and MyoG in response to external stimuli, coordinated by PASK [32,113]. PASK is a kinase predominantly found in stem cells [114]. It possesses both a sensory and a catalytic domain that help it orchestrate cellular responses to environmental stimuli [114]. Within the muscle context, PASK plays a distinctive role in mediating communication between glutamine signaling and epigenetic promyogenic alterations [32,113,114]. For this process to occur, PASK must be acetylated by p300 to accumulate in the nucleus, while also phosphorylated by mTORC1 to enhance its activity towards WDR5 [32,113,114]. Both signals are directly mediated by glutamine stimulus [113,117]. Notably, this glutamine stimulus is not endogenously produced by muscle cells themselves but is instead secreted by inflammatory macrophages [117].

**Figure 4.**
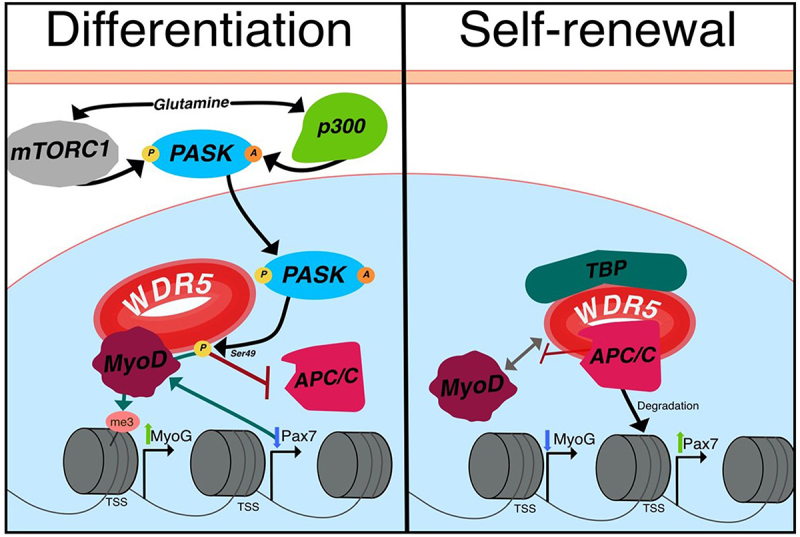
Regulation of SC differentiation or self-renewal based on WDR5 phosphorylation. In response to glutamine stimulation by macrophages, p300 acetylates and mTORC1 phosphorylates PASK, allowing its entry into the nucleus and subsequent phosphorylation of WDR5 at Ser49. This phosphorylation inhibits interactions with APC/C, leading to decreased Pax7 expression. In contrast, phosphorylation enhances WDR5’s interaction with MyoD and facilitates H3K4me3 deposition at the MyoG TSS. Additionally, the reduction in Pax7 levels further enhances MyoD expression. Conversely, in the absence of glutamine stimulation during self-renewal, WDR5 forms a complex with TBP and APC/C, promoting the rapid degradation of histones around the Pax7 TSS after mitosis, thereby enhancing its expression once the cell cycle resumes.

Once PASK is activated, it phosphorylates WDR5 at Ser49 [32]. This phosphorylation disrupts the interaction between WDR5 and APC/C [113]. As previously mentioned, the WDR5-APC/C interaction controls self-renewal by degrading histones within the TSS of pluripotency genes during mitosis, ensuring their expression in the subsequent cell cycle [63]. Although not directly studied, it appears that WDR5-APC/C also regulates Pax7 within this mechanism. As the inhibition of APC/C or the phosphorylation of WDR5 reduces the population of Pax7+ cells, contrary to the inhibition of PASK, which increases it [32,113]. In this way, the loss of Pax7 should be sufficient to promote SC differentiation through the upregulation of MyoD [118]. Interestingly, phosphorylation of WDR5 is also required for the subsequent upregulation of MyoG by MyoD [32]. WDR5 Chromatin Immunoprecipitation (ChIP) assays have demonstrated a heightened concentration of WDR5 at the TSS of MyoG following 1 day of C2C12 differentiation [32]. This enhanced concentration of WDR5 comes with a proportional increase in H3K4me3 and MyoD within MyoG [32]. In this manner, WDR5 emerges as a pivotal regulator of both SC self-renewal and myogenesis, with the interplay between these roles mediated by glutamine signaling through PASK phosphorylation (Figure 4). This underscores the significance of WDR5 in muscle regeneration and emphasizes the importance of PTMs in modulating WDR5 activity.

### WDR5 and MLL1/2

**Figure 5.**
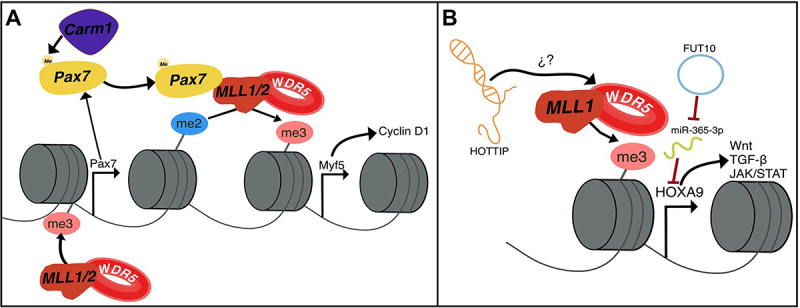
WDR5-MLL1/2 roles in skeletal muscle. A) Putative role of the WDR5-MLL1/2 complex in SC differentiation. MLL1/2 regulates Pax7 expression through H3K4me3 deposition. Subsequently, Carm1-mediated methylation of Pax7 enables its interaction with MLL1/2, facilitating the conversion of H3K4me2 to H3K4me3 at the Myf5 locus. This modification leads to the upregulation of cyclin D1 expression, promoting SC differentiation. B) WDR5-MLL1/2 dysregulation in aging SCs. Abnormal WDR5/MLL1 levels in aging SCs result in excessive H3K4me3 deposition at Hoxa9, leading to its upregulation and the activation of pathways such as Wnt, TGFβ, and JAK/STAT. Additionally, miR-365-3p is known to regulate Hoxa9; however, in aging SCs, FUT10 sequesters miR-365-3p, preventing Hoxa9 inhibition. Furthermore, the possible role of HOTTIP in guiding the MLL1/WDR5 complex is highlighted.

WDR5 is also involved in other myogenesis mechanisms as part of the WRAD/HMT complexes. The earliest documented interaction is with WRAD/MLL2 and PAX7 (Figure 5(A)) [27]. As previously mentioned, PAX7 is a key regulator for SC self-renewal, and within committed SCs, it appears to induce myogenic programming by influencing downstream targets, one of which is Myf5 [27,119]. It has been reported that Pax7+/Myf5+ cells are committed SCs, whereas Pax7+/Myf5− satellite cells contribute to the SC pool [120]. Moreover, Pax7 regulates the expression of Myf5 by binding to H3K4me2 marks within the TSS region and recruiting WRAD, which promotes H3K4me3 and Myf5 transcription (Figure 5(A)) [27]. The HMT responsible for the transition from H3K4me2 to H3K4me3 can be either MLL2 or MLL1 [27,86]. The mechanism that mediates Pax7’s role within asymmetric division involves Carm1 mediated arginine methylation of Pax7, which allows both MLLs to interact with Pax7 and induce Myf5 expression during asymmetric division [121]. However, WDR5 might also play a significant role in this mechanism due to its known role in recognizing H3K4me2 [37,45,46], and to promote the conversion to H3K4me3 [48]. Moreover, this mechanism resembles the recruitment of WDR5 to regions with H3K4me2 by lncCARs [48].

Furthermore, MLL1 has also been shown to regulate Pax7 expression, promote self-renewal, and support myogenesis in committed SCs (Figure 5(A)) [86,122]. Addicks et al. demonstrated that the conditional knockout (cKO) of MLL1 in myoblasts led to the loss of Pax7 and Myf5, as well as reduced proliferation [86]. Interestingly, in this knockout model, the authors reported no change in myoblast differentiation potential when cells were plated at a high confluency, also MyoG and Myosin Heavy Chain (MHC) expression was detected [86]. However, in vivo, there was a decrease in MyoG-expressing cells [86]. Conversely, Cai et al. used siRNA against MLL1, which also resulted in the loss of Myf5 and decreased proliferation, but in this case, it affected myogenic differentiation [122]. This suggests that MLL1 is crucial for controlling myoblast proliferation through Cyclin D1, a deficit that can be rescued by Myf5 expression (Figure 5(A)) [122]. However, MLL1’s role in differentiation appears more controversial. The difference between these observations is that Addicks used primary myoblasts, while Cai used C2C12 cells. Additionally, Addicks’ in vivo results resemble Cai’s observations with C2C12 cells. Moreover, it is noteworthy that WDR5 is needed for MyoD-induced MyoG expression through H3K4me3 [32]. And that MLL1 has been shown to be completely dependent on WDR5 for its function [74], this should indicate that WDR5 is indeed required for myoblast differentiation.

Recent single-cell analysis of murine muscular dystrophy models (mdx and D2-mdx) reveal satellite-cell subpopulations with stalled differentiation, increased senescence and apoptosis [123]. These transcriptional defects are consistent with a failure to establish or maintain H3K4me3 at key myogenic and homeostatic genes, suggesting that loss of WRAD/MLL activity could contribute to stem-cell dysfunction and disease progression, exacerbating the regenerative deficit in dystrophic muscle [27,124]. Furthermore, reduced H3K4me3 has been linked to senescence and apoptosis in multiple cellular contexts, supporting a causal connection between epigenetic deregulation and cellular aging [124,125].

Beyond dystrophies, WDR5 and MLL1 have shown to be related with muscle aging [126]. This by promoting the expression of Hoxa9, which decreases the number of Pax7+ SCs and overall muscle regeneration in aged mice [126]. This occurs by activating signaling pathways such as Wnt, TGFβ, and JAK/STAT (Figure 5(B)) [126], ultimately resulting in impaired but reversible SC differentiation and proliferation [126,127]. As the inhibition or silencing of WDR5 or MLL1 decreased Hoxa9 expression and improves muscle regeneration [126]. The increase in Hoxa9 within aged SCs appears to be more regulated by WDR5 location, as both WDR5 and H3K4me3 are highly enriched at the Hoxa9 locus [126]. Additionally, this phenomenal have also seen within muscle denervation [127]. In denervated muscle, Hoxa9, WDR5 and MLL1 are found upregulated [127], in which, Hoxa9 directly promotes atrophic signaling, and SC apoptosis [127].

The abnormal localization of WDR5 and dysregulation of Hoxa9 is intriguing. One mechanism for this is the upregulation of circRNA FUT10, which sponges miR-365-3p, a known inhibitor of Hoxa9 expression (Figure 5(B)) [128]. However, this does not explain the abnormal localization of WDR5. To date, WDR5 localization appears to be partially regulated by lncRNAs [48,57]. It is well known that the lncRNA HOTTIP upregulates Hoxa9 expression through WDR5 [105,129,130]. Thus, HOTTIP is a strong candidate to explain the abnormal localization of WDR5 near the Hoxa9 locus. However, as of now, HOTTIP has not been studied within adult skeletal muscle (Figure 5(B)).

### WDR5 and lncRNA within muscle

**Figure 6.**
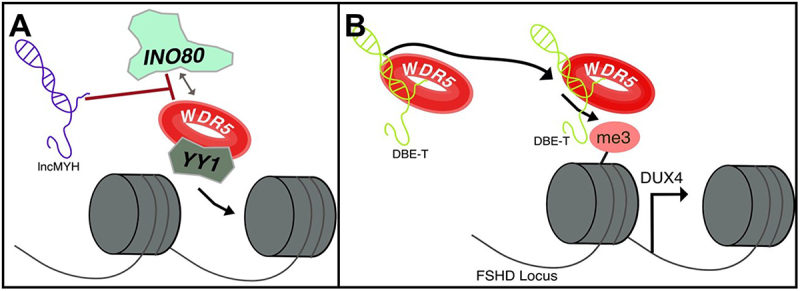
Known roles of lncRNA complexes in skeletal muscle. A) LncMYH inhibits the formation of the INO80-WDR5-YY1 complex in SCs, preserving their quiescent state. B) LncRNA DBE-T guides WDR5 to the FSHD locus, enhancing H3K4me3 deposition and promoting Dux4 expression.

A highly conserved long non-coding RNA, lncMYH, transcribed from the fast MYH locus [131], has been shown to inhibit the recruitment of WDR5 and YY1 to the INO80 complex in SCs, thereby helping to maintain their quiescent state (Figure 6(A)). In contrast, lncMYH knockout leads to increased WDR5-INO80 interaction promoting SC proliferation [132]. This finding is particularly interesting because previous studies describe lncRNA as scaffolds that guide WDR5 to specific genomic loci, but lncMYH appears to interfere with WDR5 complex formation, representing a novel regulatory mechanism. Although the molecular details of the WDR5-INO80 interaction remain unclear this interaction has also been observed during osteogenic differentiation where both WDR5 and INO80 upregulate the Wnt signal and support specific cell type program [59]. Notably, Schutt et al. (2020) couldn’t detect any MLL protein associated with the INO80 complex, suggesting that WDR5 can form functional complexes independently of MLL. Collectively, this evidence supports a model in which lncMYH regulated the quiescence-to-activation transition in skeletal muscle SCs by modulating WDR5-INO80 complex formation.

WDR5 has also been reported to contribute to the pathological development of FSHD [61], a neuromuscular disease marked by the death and deterioration of muscle fibers, resulting in increasing muscle weakness and wasting [133]. FSHD is directly linked to epigenetic changes within the D4Z4 loci, resulting in the loss of repression markers such as DNA methylation and H3K9me3 or H3K27me3 [61,133]. This ultimately leads to the abnormal expression of the DUX4 gene, which is mainly found during early development [61,133]. This aberrant expression of DUX4 inhibits myogenesis and induces apoptosis [61,134]. Also DUX4 is highly regulated in cis by the lncRNA DBE-T [61]. Interestingly, Mocciaro et al. (2023) found through mass spectrometry that WDR5 is a key partner of DBE-Tc [61]. Moreover, DBE-T recruits WDR5 to the FSHD locus, directly upregulating DUX4 expression (Figure 6(B)) [61]. Finally, silencing WDR5 or inhibiting its WIN site pharmacologically rescues FSHD muscle cell differentiation and viability [61]. Once again, this highlights the intrinsic role of lncRNA in guiding WDR5 through the genome, ultimately leading to transcript activation.

As previously observed in melanoma, the long noncoding RNA NEAT1 has been shown to sequester WDR5 and BRD4 into repressive complexes, highlighting its role as an epigenetic regulator [135]. In skeletal muscle, NEAT1 is overexpressed in glucocorticoid-induced atrophy [136]. Also, in the context of myogenesis, an RNA pull-down of NEAT1 identified several functional interactors [137]. Notably, WDR5 and BRD4 were not detected in this pull-down, likely due to technical limitations, as the mass spectrometry analysis was performed only on proteins excised from differentially stained gel bands.

Nonetheless, it remains plausible that under specific physiological or stress conditions, NEAT1 could engage WDR5 and BRD4 in skeletal muscle, potentially modulating transcription of myogenic and contractile genes. This hypothesis is indirectly supported by recent evidence that BRD4 regulates myogenesis [137].

## WDR5 on smooth muscle

**Figure 7.**
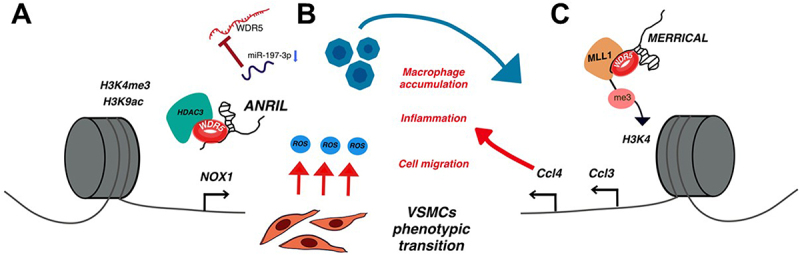
Known roles of WDR5 in smooth muscle. A) WDR5 regulates key SMC marker genes through the WDR5/Pitx2 complex, enhancing H3K4me3 deposition at SM α-actin and SM22α, promoting their expression. B) WDR5 contributes to atherosclerosis in smooth muscle. The HDAC3-ANRIL-WDR5 complex facilitates both H3K9ac and H3K4me3 modifications, leading to NOX1 upregulation, promoting VSMCs phenotypic transition and increasing reactive oxygen species (ROS). Additionally, miR-197-3p, a known regulator of WDR5, is downregulated in atherosclerosis patients. C) the MLL1-WDR5-MERRICAL complex drives the expression of pro-inflammatory genes such as Ccl3 and Ccl4 in macrophages, contributing to atherosclerosis.

Similar to its role in skeletal muscle, WDR5 regulates smooth muscle cell (SMC) differentiation [29]. Research indicates that knocking down WDR5 decreases the expression of key SMC marker genes, such as SM α-actin and SM22α, during in vitro SMC differentiation [29]. This regulatory mechanism involves the interaction of the WRAD complex with the transcription factor Pitx2 (Figure 7(A)) [29]. Furthermore, both WDR5 and Pitx2 knockouts lead to reduced H3K4me3 levels at the promoter regions of SM α-actin and SM22α [29]. Also, alterations at the Pitx2 binding site, ATTA cis element, result in lower H3K4me3 levels suggesting that WDR5 is recruited to these gene loci in a Pitx2-dependent manner [29]. Collectively, these findings highlight a critical role for WDR5 in maintaining SMC identity, in line with observations that lncRNAs or other proteins can regulate WDR5 localization.

Furthermore, vascular smooth muscle cells (VSMCs) exemplify the functional consequences of WDR5 regulation in a highly plastic cellular context. VSMCs can switch from a contractile state under basal conditions to a proliferative, migratory phenotype during tissue injury [99]. This phenotypic switch is regulated, in part, by the lncRNA NEAT1, which is upregulated in VSMCs following vascular injury in vivo and in vitro. NEAT1 sequesters WDR5 and prevents its epigenetic function. As a result, the deposition of the H3K4me3 mark is reduced, leading to the downregulation of smooth muscle – specific contractile genes [99,138].

Consequently, WDR5 is increasingly recognized as a pivotal factor in the context of atherosclerosis, a chronic progressive inflammatory disease characterized by arterial plaque formation [139,140]. These plaques are formed by lipids, cells, debris, and scar tissue, and its presence leads to severe clinical consequences such as myocardial infarction and strokes [139,140]. VSMCs play a crucial role in this disease, undergoing a phenotypic switch under pathological conditions characterized by increased migration and enhanced proliferation rates [139,140]. This transition promotes plaque progression and stabilization [62,139,140]. WDR5 mediates this phenotypic transition through its interaction with a lncRNA called ANRIL [62]. ANRIL is upregulated in patients with coronary atherosclerotic heart disease [62]. Moreover, it guides and stabilizes the HDAC3-WDR5 complex at the NOX1 promoter, enhancing H3K4me3 and H3K9ac active marks that upregulate NOX1 expression (Figure 7(B)) [62]. Thus, in this scenario, the lncRNA not only regulates WDR5 localization but also its interactions.

Moreover, WDR5 inhibition has demonstrated therapeutic potential in treating atherosclerosis [141]. Yang et al. observed that miR-197-3p is downregulated in patients with atherosclerosis and that its ectopic expression in vitro inhibits VSMC proliferation and migration (Figure 7(B)) [141]. Furthermore, WDR5 was identified as a target of miR-197-3p, with WDR5 overexpression reversing the effects of miR-197-3p in vitro [141]. Additionally, WDR5 is implicated in regulating the inflammatory response in diabetes-associated atherosclerosis through its interaction with the lncRNA MERRICAL, expressed within macrophages [60]. Mechanistically, MERRICAL guides the WDR5-MLL1 complex to activate transcription of CCL3 and CCL4 via H3K4me3 modification, promoting a pro-inflammatory state (Figure 7(C)) [60]. Thus, WDR5 emerges as a key regulator in atherosclerosis, mediating VSMC transition and inflammatory processes.

The significant role of WDR5 in atherosclerosis is notably intriguing, particularly given its link to sarcopenia. Recent insights suggest a potential relationship between sarcopenia and atherosclerosis, mediated through dysregulated release of myokines and other factors influencing the body’s secretome [142,143]. In 2020, Campos et al., investigated this association within 208 individuals with at least 80 years and without prior cardiovascular diseases [142]. They found an association between subclinical atherosclerosis and the reduction of muscle mass and strength through sarcopenia [142]. In the same year Uchida et al. studied 321 patients with at least 65 years and ischemic heart disease [143]. They found that atherosclerosis was associated with a loss of muscle function of the lower limbs [143]. Additionally, atherosclerosis, as sarcopenia, appears to be regulated by HOXA9 [Fu, et al., 2023]. In this context, HOXA9 mediates VSMC phenotypic switching by upregulating Methyl-CpG binding protein 2 (MECP2) expression [144]. Although WDR5’s specific role has not been studied, its involvement in HOXA9 regulation in skeletal muscle makes it a compelling candidate for HOXA9 regulation in VSMC phenotypic switching. Furthermore, the potential regulation of HOXA9 by HOTTIP, which has been shown to guide WDR5 within the HOXA cluster, warrants further investigation, as its role in both pathologies remain unclear.

## Inhibitors of WDR5

WDR5 functions primarily as a molecular hub, orchestrating multiprotein assemblies through two distinct binding interfaces: the WIN site and the WBM site. This dual architecture offers multiple avenues for therapeutic intervention, giving rise to three main strategies: direct blockade of the WIN site, disruption of the WBM site, and targeted degradation of WDR5 via PROTACs [145].

Among these strategies, efforts to target the WIN site have progressed the furthest. This pocket recognizes a conserved arginine within the WIN motif of MLL/SET family proteins, creating a precise molecular hotspot for small-molecule design. Early approaches employed peptidomimetics based on the minimal MLL1 recognition sequence; compounds such as MM-102 demonstrated strong binding affinity and clarified the structural basis of WIN-site engagement. However, these peptide-like molecules suffered from poor cellular permeability, limiting their practical application [145].

The development of OICR-9429, a piperazine-based inhibitor with nanomolar affinity, represented a major advance. By disrupting WRAD complex formation in cells, OICR-9429 quickly became a benchmark chemical probe. Subsequent studies have produced additional small molecules with sub-nanomolar potency and improved pharmacokinetic profiles [146–151].

Despite these advances, a key challenge remains, reconciling the biological effects of genetic versus pharmacological WDR5 disruption. Complete genetic knockout leads to global loss of H3K4me3 and widespread gene dysregulation, whereas WIN-site inhibition has a narrower impact, preferentially downregulating defined target transcripts [145]. Guarnaccia et al. (2021) used quantitative proteomics to show that WIN-site inhibitors largely affect canonical WDR5 partners, such as MLL and NSL complexes, while a small subset associated with replication, transcription, and chromatin remodeling became enriched. This highlights the potential of WIN-site inhibitors’ ability to modulate non-canonical WDR5 functions [152].

Siladi et al. (2022) compared acute WDR5 degradation with WIN-site inhibition, revealing that WDR5 loss broadly impacts the transcriptome, whereas WIN-site inhibitors only affect a subset of these genes. Further analysis confirmed that WDR5 directly regulates transcription at thousands of loci independently of H3K4me3 deposition. These findings explain the therapeutic selectivity of WIN-site inhibitors and underscore the genome-wide transcriptional reprogramming triggered by WDR5 loss [153]. Which opens the possibility of not only targeting WDR5 for a total loss of its function, but to modulate its ability and chromatin remodeling potential.

Complementing site-specific inhibition, targeted degradation of WDR5 using PROTACs offers a strategy for more complete functional ablation. These bifunctional molecules link a protein of interest to an E3 ubiquitin ligase, triggering ubiquitination and proteasomal degradation in a catalytic repeatable manner. Early WDR5 PROTACs incorporated known inhibitors, such as OICR-9429 and pyrroloimidazole-based scaffolds, paired with representative E3 ligases [12]. Li et al. (2022) reported MS40, a cereblon (CRBN)-recruiting PROTAC that selectively degrades WDR5, inducing the disassemble of the MLL complex from chromatin, decreasing H3K4me2 and repressing transcription of WDR5 -dependent targets [147]. More recently, Mabanglo et al. (2024) developed DCAF1-recruiting PROTACs targeting WDR5, identifying four compounds that degrade WDR5 in cells. Structural studies of ternary complexes revealed how DCAF1 recognizes WDR5-PROTACs, providing guidance for designing more stable and effective degraders [154].

In parallel, the shallower WBM site, which mediates interactions with MYC and RbBP5, has emerged as a compelling therapeutic target. Unlike the more accessible WIN site, the WBM site is inherently less druggable, posing challenges for inhibitor development [145,155]. Despite these obstacles, structure-based approaches have produced lead compounds capable of selectively disrupting WDR5:MYC and WDR5:RbBP5 interactions [155]. Notably, compound 7k dose-dependently inhibits these interactions, making it a widely used and valuable tool in research [156]. For instance, beyond protein – protein interactions, 7k have also been used to modulate WDR5-lncRNA complexes. For instance, it disrupts HOTTIP – WDR5 and HOXC13-AS – WDR5 assemblies, leading to downregulation of HOTTIP in MDA-MB-231 cells [157]. Which is consistent with the ability of some lncRNAs to regulate their own expression through WDR5 interactions, as previously discussed. However, FOXD3-AS1 is insensitive to 7k, further highlighting differences in lncRNA target responsiveness and suggesting potential variability in WDR5 interaction sites. But also highlighting the potential of some inhibitors modulating specific WDR5-lncRNA complexes and its role on WDR5 location within the chromatin.

Overall, the outlook for WDR5-targeted therapeutics highlights that, although WDR5 activity is governed primarily by two key regions, blocking a single site is generally insufficient to abrogate all WDR5 functions. Literature suggests that small-molecule inhibitors may not fully occupy these sites but instead modulate WDR5 interactions with specific partners, offering opportunities to selectively fine-tune activity. For cases where complete elimination of WDR5 is desired, PROTAC-based degraders provide a complementary strategy. The structural architecture of WDR5 thus offers a versatile platform for developing molecules that act on canonical or less-explored pockets within both binding interfaces, expanding therapeutic possibilities.

## Opportunities and open questions

To this point, it is clear that WDR5 functions as a master regulator across multiple chromatin-associated complexes. Its two primary binding interfaces, WIN and WBM, provide versatile docking points that connect diverse partners and assemblies. Within the canonical WRAD complex, WDR5 serves as an anchor, bridging nucleosome-bound RbBP5 and members of the SET1/MLL family, thereby enabling H3K4me3 deposition [66]. Beyond this catalytic role, WDR5 also acts as a reader of histone modifications, recognizing marks such as H3R2me2s, H3Q5ser, H3K4me2, and H3T11P (Table 1). This recognition capacity allows WDR5 to interpret chromatin cues and recruit appropriate complexes. In addition to WRAD, WDR5 is integrated into NuRD, HDACs, NSL, and other assemblies, suggesting that each binding surface can selectively recruit distinct partners (Table 1). Over time, WDR5 will likely be identified in even more complexes central to epigenetic regulation. This broad participation explains how WDR5 exerts both cooperative and opposing effects on chromatin structure, gene expression, and cellular states.

As WDR5 research enters a new era, a key question is whether WDR5 acts as a general scaffold for diverse complexes or is restricted and guided by specific regulatory mechanisms. Current evidence favors the latter. WDR5 is not a passive adaptor but a dynamically regulated protein whose activity is shaped by multiple layers of control. As inhibiting the WIN or WBM sites has been shown to modulate and rewire the WDR5 interactome, ensuring that WDR5 can flexibly switch between transcriptional activation, repression, chromatin remodeling, and even structural roles during mitosis [152,153].

PTMs and micropeptides represent an additional, critical layer of WDR5 regulation. Ubiquitination, mediated by FBXW7, CUL4B – DDB1, or RNF220, can target WDR5 for degradation, modulating its incorporation into specific complexes and controlling programs such as mitotic progression or Hox gene expression [101,110]. Methylation at Lys207 and Lys325 by SETD6 May influence proliferation and migration while partially maintaining H3K4me3, although its impact on complex assembly remains unclear [111]. SUMOylation of WDR5 and RbBP5 appears to regulate SET1/MLL assembly in specific contexts [112]. Phosphorylation of Ser49 by PASK alters interactions with APC/C, affecting self-renewal and mitotic gene expression [32]. Meanwhile, micropeptides such as EMBOW can shift WDR5 from an epigenetic regulator to a structural component within the mitotic spindle [64]. Together, these findings prompt key questions: which PTMs or peptides are associated with specific complexes or cellular states, and can these modifications be mimicked or blocked therapeutically? Systematic mapping and targeted modulation could allow precise control of WDR5 activity, steering it toward transcriptional activation, repression, chromatin remodeling, or mitotic scaffolding.

Just as PTMs provide dynamic control, lncRNAs add another layer of regulation, offering sequence-specific guidance that directs WDR5 to genomic loci or scaffolds distinct complexes. The mechanisms by which lncRNAs bind WDR5 remain unclear. It is unknown whether co-localization within the same subcellular compartment is sufficient, or whether specific RNA motifs, secondary structures, or cofactors are required. Resolving these questions could reveal the molecular grammar of WDR5 targeting and facilitate the design of synthetic lncRNAs capable of guiding WDR5 to genes of therapeutic interest.

The functional outcomes of lncRNA-mediated WDR5 recruitment are not fully understood. It is unclear whether this process is always tied to H3K4me3 deposition or can also facilitate the assembly of other complexes. If so, lncRNAs may act as molecular bridges, linking chromatin remodeling with transcription factor – driven programs. Such mechanisms could be particularly relevant in muscle stem cells or diseased tissue, where reprogramming transcriptional networks might enhance regeneration or suppress maladaptive responses.

This raises the question of how fundamental lncRNA – WDR5 regulation is compared to canonical layers of control, such as histone modifications or transcription factor binding. If lncRNA – WDR5 interactions form a primary regulatory axis, they may offer exceptionally precise therapeutic entry points, with fewer off-target effects than global epigenetic drugs. Synthetic lncRNAs or small molecules that emulate natural RNA guides could be designed to harness WDR5, activating regenerative programs or silencing genes that drive muscle degeneration.

These findings highlight that while WDR5 is a key hub in chromatin biology, its regulation by lncRNAs, PTMs, and micropeptides remains poorly understood. Dissecting these mechanisms will not only clarify how WDR5 integrates diverse cellular signals but also open powerful avenues for therapeutic innovation.

Altogether, WDR5 regulation represents an evolving field of research that not only advances our understanding of epigenetics but also holds promise for developing novel strategies to manipulate chromatin regulation and gene expression. Many questions also remain in understudied contexts such as skeletal muscle.

**Figure 8.**
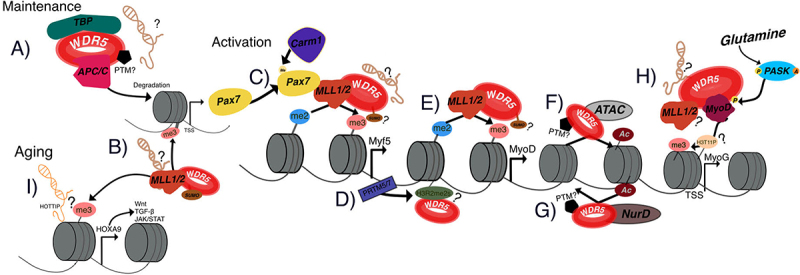
Summary of WDR5 functions in skeletal muscle. A) Association with TBP and APC/C promotes histone degradation, enabling Pax7 expression and maintenance of stemness; no PTM or lncRNA involvement has been reported. B) Participation in the WRAD – MLL1/2 complex deposits H3K4me3 at the Pax7 promoter, supporting its expression; SUMOylation may be involved, but no lncRNA association has been identified. C) Following activatory stimuli, Pax7 is methylated by Carm1, which recruits WRAD – MLL1/2 to establish H3K4me3 at the Myf5 promoter; SUMOylation may be involved, but no lncRNA association has been identified. D) PRMT5 and PRMT7 catalyze H3R2me2s, enhancing regenerative capacity and potentially facilitating WDR5 binding and subsequent H3K4me3 deposition. E) WDR5 preferentially recognizes H3K4me2, particularly in association with lncRnas. In skeletal muscle, H3K4me2 enrichment at Myf5 and MyoD primes genes for activation, suggesting WDR5 mediates the H3K4me2-to-H3K4me3 transition. F) Interaction with the ATAC complex and KAT2B promotes histone acetylation and regulates proliferation required for myogenic programs. G) Possible association with the NuRD complex, which maintains stem cell identity by repressing non-myogenic genes; disruption impairs regeneration and lineage fidelity. H) Phosphorylation of WDR5 at Ser49 by the nutrient-sensitive kinase PASK disrupts the WDR5–APC/C interaction, promoting myogenic commitment and MyoG activation via MyoD. WDR5 may also be recognizing H3T11 phosphorylation at Myog, which may act as a recruitment signal. I) mislocalization of WDR5 leads to aberrant H3K4me3 enrichment at loci such as Hoxa9, activating maladaptive Wnt, TGFβ, and JAK/STAT pathways. The lncRNA HOTTIP may contribute to this mislocalization.

Skeletal muscle regeneration depends on SCs, the adult stem cells responsible for rebuilding tissue following injury. SC fate is orchestrated by a network of transcription factors, with Pax7 maintaining stem cell identity and Myf5, MyoD, and MyoG driving commitment and differentiation (Figure 8). WDR5 is intimately involved at multiple nodes within this regulatory network. One of the best-characterized mechanisms is phosphorylation of WDR5 at Ser49 by PASK, a nutrient- and glutamine-responsive kinase [32,113]. Glutamine signaling, often provided by inflammatory macrophages in the regenerating niche, triggers PASK nuclear accumulation and activation. Once activated, PASK phosphorylates WDR5, disrupting its interaction with the APC/C complex (Figure 8(A&H)). This disassembly reduces Pax7+ stem cell populations, facilitating myogenic commitment, while simultaneously enabling MyoD-dependent activation of MyoG through WDR5 recruitment to its transcription start site. The result is a finely tuned switch: a single PTM converts WDR5 from a stem cell maintenance factor into a differentiation facilitator, highlighting the precision of post-translational control in myogenesis.

An important therapeutic avenue could be the development of molecules to either maintain SCs in a Pax7+ state or accelerate regeneration by promoting differentiation. Targeting WDR5 phosphorylation at Ser49 May allow precise temporal control of stem cell maintenance versus differentiation, offering potential interventions for muscle-wasting diseases or injury repair.

Beyond PASK-dependent regulation, WDR5 also functions as an essential scaffold for WRAD/MLL complexes in SCs [27]. Pax7 recruits WDR5 and WRAD to the Myf5 promoter, where WDR5’s ability to recognize H3K4me2 should facilitate its conversion to H3K4me3, activating transcription. Both MLL1 and MLL2 contribute to this transition, and the cooperation between Pax7 (Figure 8(B,C)), Carm1-mediated arginine methylation, and WDR5 ensures the asymmetric division of SCs, maintaining a pool of undifferentiated cells while promoting lineage commitment [121].

WDR5’s role in asymmetric cell division highlights the possibility that Ser49-phosphorylated WDR5 May be spatially restricted within this context. Understanding these mechanisms may enable manipulation of stem cell fate at the single-cell level to enhance skeletal muscle regeneration.

Loss of WRAD/MLL activity, whether through knockout of MLL1 or mislocalization of WDR5, compromises SC proliferation and differentiation, as observed in aged or dystrophic muscle. In these contexts, aberrant enrichment of WDR5 and H3K4me3 at loci such as Hoxa9 promotes maladaptive signaling through Wnt, TGFβ, and JAK/STAT pathways, ultimately impairing regeneration [126]. Given the known role of HOTTIP in regulating this locus, it may also explain the abnormal localization of WDR5 near Hoxa9 (Figure 8(I)). However, this hypothesis has not yet been tested, and clarifying this connection could open the door to more precise therapies targeting Hoxa9-driven aging.

Although no clear evidence has been established, several potential mechanisms suggest additional roles for WDR5 in muscle. For instance, WDR5’s capacity to interpret H3R2me2s may place it downstream of protein-arginine methyltransferases such as PRMT5 and PRMT7, which promote H3R2me2s and contribute to SC regenerative capacity [80–82]. This raises the possibility that WDR5 acts as a key effector of arginine methylation pathways during myogenesis (Figure 8(D)).

Additionally, WDR5 preferentially recognizes H3K4me2, particularly in association with lncRNAs. In skeletal muscle, regions enriched for H3K4me2 mark genes such as Myf5 and MyoD for activation during SC commitment, implying that WDR5 May facilitate the H3K4me2-to-H3K4me3 transition, thereby priming myogenic transcription. This would also suggest that lncRNAs guide WDR5 to these regions (Figure 8(E)).

Beyond methylation, WDR5 also recognizes H3T11 phosphorylation H3T11P. At promoters of myogenic genes such as Myog and Myh3, this modification may serve as a signal for WDR5 recruitment [88]. By interpreting H3T11P, WDR5 could help recruit SET1/MLL complexes and promote H3K4 methylation, thereby coupling metabolic cues to epigenetic remodeling (Figure 8(H)).

WDR5 further contributes to muscle-specific chromatin remodeling through its interactions with multiple complexes. It associates with the NuRD complex, which maintains SC identity by repressing non-myogenic genes and ensuring proper lineage progression. Disruption of NuRD components impairs regeneration and leads to lineage infidelity [93], which opens the door to WDR5 helping NuRD to modulate NuRD maintain chromatin accessibility during regeneration (Figure 8(G)). WDR5 also interacts with the ATAC complex and KAT2B, both critical for transcriptional regulation. Through these interactions, WDR5 May coordinate acetylation and methylation dynamics (Figure 8(F)), regulating proliferation genes such as Ccna2 and Cdc25c in SCs and integrating metabolic and developmental signals to fine-tune myogenic programs [98].

In smooth muscle, WDR5 also emerges as a critical regulator of phenotypic plasticity, balancing contractile identity with proliferative remodeling. This switch underlies both physiological adaptation and pathological processes such as vascular injury, restenosis, and hypertension. While transcription factors and signaling pathways have been studied in this context, the specific role of WDR5 is only beginning to be appreciated.

A central question is whether distinct lncRNA – WDR5 interactions guide its recruitment to contractile versus proliferative gene programs. If so, targeting these RNA – protein interfaces could provide strategies to preserve vascular stability while maintaining the capacity for repair. Equally important is understanding how WDR5 integrates local chromatin remodeling with systemic processes such as inflammation, atherosclerosis, or sarcopenia. By bridging transcriptional control with environmental cues, WDR5 May act as a molecular link coupling smooth muscle remodeling to organismal physiology.

Finally, the interaction of WDR5 with HOXA9 and its lncRNA regulator HOTTIP raises the possibility of a unifying mechanism across tissues. Mislocalization of WDR5 near HOXA9 has been implicated in skeletal muscle degeneration, and a similar mechanism could contribute to smooth muscle dysfunction in vascular disease. Exploring whether these interactions converge on common pathways of maladaptive remodeling could reveal new entry points for therapeutic intervention.

Together, these insights position WDR5 as more than a structural adaptor: it is a dynamically regulated hub that integrates chromatin cues, RNA guidance, and post-translational modifications to govern cell fate and tissue function. Yet the full scope of its regulation remains unresolved. Addressing these questions will not only clarify how WDR5 navigates between competing programs but also reveal whether its versatility can be harnessed for therapy. Ultimately, dissecting the regulatory logic of WDR5 May enable the design of interventions that preserve stem cell potential, restore youthful muscle function, and prevent maladaptive remodeling, placing WDR5 at the center of future epigenetic medicine.

## Conclusions

WDR5 emerges as a remarkably versatile protein that integrates multiple layers of cellular regulation. Beyond its well-established role in epigenetic regulation via H3K4 methylation as part of the WRAD/SET1-MLL complexes, it is now clear that WDR5’s functional landscape extends far beyond this. It participates in both acetyltransferase and deacetylase complexes, is subject to fine-tuned regulation by long non-coding RNAs (lncRNAs), microproteins, and diverse post-translational modifications (PTMs), serving as a scaffold for numerous chromatin-associated machineries.

In skeletal muscle, WDR5 exemplifies this regulatory complexity. Phosphorylation by PASK coordinates the activation of Pax7 and MyoG, balancing satellite cell (SC) self-renewal and differentiation. Meanwhile, lncRNAs such as lncMYH modulate WDR5’s interaction with the INO80 complex, maintaining SC quiescence and safeguarding the stem cell pool. These findings underscore WDR5’s central role in governing muscle homeostasis and regeneration, acting as a molecular hub where signaling pathways converge to control fate decisions.

Beyond muscle biology, WDR5 has critical functions in stem cell maintenance, pluripotency, and lineage specification, highlighting its widespread relevance in development and disease. Yet, despite substantial progress, significant gaps remain. The dynamic composition of the WDR5 interactome across cellular states such as differentiation, regeneration, and tumorigenesis remains poorly mapped. Additionally, the context-specific impact of its PTMs and lncRNA-mediated regulation is still largely uncharacterized.

Future studies that explore these regulatory mechanisms may reveal fundamental principles of chromatin control and cellular plasticity. Given its modular structure and central role in epigenetic networks, WDR5 emerges as both a key integrator of chromatin-based signaling and a promising therapeutic target in muscle degenerative diseases, cancer, and regenerative medicine.

## Publicly available data

This article does not require or generates any new data for publication.
